# Acute Hepatic Porphyria: Pathophysiological Basis of Neuromuscular Manifestations

**DOI:** 10.3389/fnins.2021.715523

**Published:** 2021-09-27

**Authors:** Paulo Victor Sgobbi de Souza, Bruno de Mattos Lombardi Badia, Igor Braga Farias, Wladimir Bocca Vieira de Rezende Pinto, Acary Souza Bulle Oliveira

**Affiliations:** Division of Neuromuscular Diseases, Department of Neurology and Neurosurgery, Federal University of São Paulo (UNIFESP), São Paulo, Brazil

**Keywords:** acute hepatic porphyria, neuromuscular, neuropathy, rhabdomyolysis, dysautonomia, pathophysiology, inherited metabolic diseases, inborn errors of metabolism (IEM)

## Abstract

Acute hepatic porphyria represents a rare, underdiagnosed group of inherited metabolic disorders due to hereditary defects of heme group biosynthesis pathway. Most patients have their definite diagnosis after several years of complex and disabling clinical manifestations and commonly after life-threatening acute neurovisceral episodes or severe motor handicap. Many key studies in the last two decades have been performed and led to the discovery of novel possible diagnostic and prognostic biomarkers and to the development of new therapeutic purposes, including small interfering RNA-based therapy, specifically driven to inhibit selectively delta-aminolevulinic acid synthase production and decrease the recurrence number of severe acute presentation for most patients. Several distinct mechanisms have been identified to contribute to the several neuromuscular signs and symptoms. This review article aims to present the current knowledge regarding the main pathophysiological mechanisms involved with the acute and chronic presentation of acute hepatic porphyria and to highlight the relevance of such content for clinical practice and in decision making about therapeutic options.

## Introduction

**FIGURE 1:**
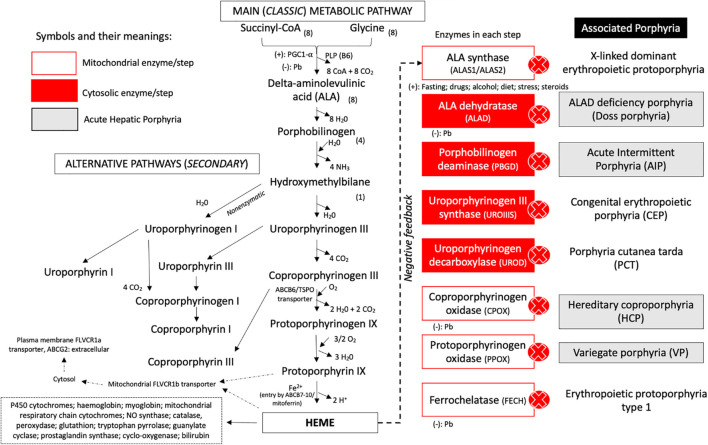
Metabolic pathways and steps of heme group biosynthesis and correlation of enzyme deficiency and associated porphyria. The classic and alternative pathways associated with heme biosynthesis are presented with the specific transporters, cofactors, and reagents involved in each chemical reaction. The enzymes involved in each step of biosynthesis are represented at the right side of the metabolic route. After the heme group has been produced, it promotes downregulation of ALA synthase and can be incorporated by mitochondrial hemoproteins or leave the mitochondrial compartment by the FLVCR1b transporter and come to the cytoplasm to be incorporated by several compounds. In the plasma membrane, the ABCG2/FLVCR1a transporter is present and can regulate the efflux of the heme group. Acute hepatic porphyria (AHP) types are presented in gray boxes. Cytosolic steps are represented in red boxes, and intramitochondrial enzymes are presented in white boxes. Legend: ALA, delta-aminolevulinic acid; CoA, coenzyme A; NO, nitric oxide; (–), inhibition (downregulation) of the enzymatic step; (+), upregulation (stimulation) of the step.

Porphyrias (from the Greek *porphyrus*, purple) are a rare group of inherited or acquired metabolic disorders of the heme biosynthesis pathway, which has eight biochemical steps (Figure 1) with abnormal production and accumulation of toxic intermediate metabolites, which enter in the blood circulation and are excreted into the urine or bile (Bissell et al., 2017; Souza et al., 2021).

Heme group production occurs predominantly in erythroblasts from the bone marrow (almost 80% of total heme production) and liver (around 20% of total heme group) and plays a major role as cofactor of different iron-based hemeproteins, such as hemoglobin, hepatic cytochromes P450, myoglobin, mitochondrial respiratory chain cytochromes, some catalases and peroxidases, and microsomal cytochrome b5 (Bissell et al., 2017; Wang et al., 2018; Phillips, 2019; Souza et al., 2021).

The biochemical synthesis pathway involves eight enzymatic reactions dependent on several cofactors, located in the cytoplasm or mitochondrial compartments and modulated by genetic modifiers and individual high-susceptibility risk factors (i.e., cytochrome P450-coding genes and their polymorphisms), endocrine (i.e., menstrual cycle, pregnancy, and puerperium), environmental (i.e., lead poisoning), dietetic factors (i.e., alcohol consumption, prolonged fasting or low carbohydrate diet, and cigarette smoking), and unsafe porphyrinogenic drugs (i.e., hormonal contraceptives, phenytoin, barbiturates, and sulfonamides) (Phillips, 2019; Souza et al., 2021). The rate-limiting step in this pathway is performed by the ubiquitous housekeeping isozyme delta-aminolevulinic acid synthase 1 (ALAS1) in the liver and by the erythroid tissue-specific isozyme ALAS2 in bone marrow, induced directly or indirectly by precipitating factors or triggers (Besur et al., 2014; Stojanovski et al., 2019; Souza et al., 2021; Figure 1).

**FIGURE 2:**
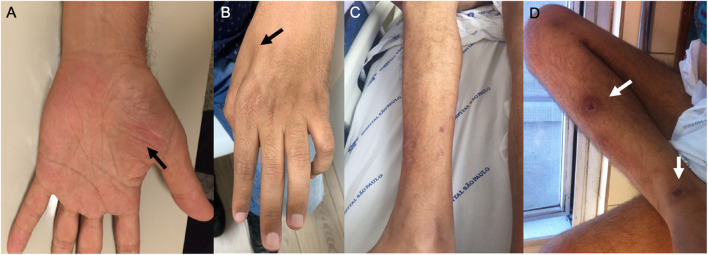
Examination findings in patients with AHP. **(A)** Amyotrophy of the tenar region (black arrow) and **(B)** severe amyotrophy of the first dorsal interosseous muscle of the hand (black arrows) in patients with variegate porphyria. **(C,D)** Distal amyotrophy and photosensitivity lesions in the lower limbs (white arrows) in patients with hereditary coproporphyria.

Traditionally, the porphyrias have been classified according to the main tissue in which heme precursors accumulate as hepatic or erythropoietic porphyrias, and based on clinical manifestations as acute porphyrias characterized by neurovisceral attacks with abdominal pain and neurologic features or chronic porphyrias with prominent cutaneous involvement in photoexposed skin areas due to overproduction of photosensitizing porphyrins (Figure 2; Bissell et al., 2017; Wang et al., 2018; Phillips, 2019; Souza et al., 2021).

Acute hepatic porphyrias (AHPs) represent an important group of autosomal dominant or recessive inherited metabolic diseases, involving different monogenic defects of the heme biosynthesis pathway, with marked overproduction of porphyrins and their precursors in the liver leading to abnormal accumulation of intermediate metabolites, mainly porphobilinogen (PBG) and delta-aminolevulinic acid (ALA), and direct heme group deficiency (Besur et al., 2014; Souza et al., 2021).

Four distinct clinical, genetic, and metabolic AHP presentations are currently recognized: (i) acute intermittent porphyria (AIP) caused by porphobilinogen deaminase enzyme deficiency due to *HMBS* genetic variants, (ii) variegate porphyria (VP) caused by protoporphyrinogen oxidase enzyme deficiency with *PPOX* genetic variants, (iii) hereditary coproporphyria (HCP) with coproporphyrinogen oxidase enzyme defect related to pathogenic variants in *CPOX* gene, and (iv) ALA dehydratase deficiency porphyria (also known as ALAD deficiency or Doss porphyria) with abnormal enzyme activity of ALA dehydratase coded by *ALAD* gene (Bissell et al., 2017; Souza et al., 2021). Patients can rarely present with compound heterozygous or homozygous variants in the *HMBS*, *PPOX*, and *CPOX* genes with clinical manifestations presenting during infancy and including global developmental delay, failure to thrive, leukoencephalopathy, and other central nervous system abnormalities (Bonkovsky et al., 2019; Souza et al., 2021).

The AHP are a group of rare and probably underestimated disease with a global estimated prevalence of five cases per 100,000 worldwide. There are some higher prevalent regions, such as northern Scandinavia and southeast of Spain due to genetic founder effects, with recent studies showing that disease-causing variants on *HMBS* gene may be as frequent as six per 1,000 among Caucasian individuals (Chen et al., 2016; Bonkovsky et al., 2019; Souza et al., 2021).

Thus, we will present and discuss in this article the main pathophysiological mechanisms associated with neuromuscular manifestations of AHP and correlate the potential implications of the use of new therapeutic purposes for acute, recurrent, and chronic contexts.

## Clinical Overview of Acute Hepatic Porphyrias

Nowadays, the AHP are considered a chronic condition with a negative impact on physical and emotional health, leading to low quality of life and complicated by acute attacks, which typically present with multiple dysfunctions of autonomic, peripheral, and central nervous system, requiring hospitalization and not rarely with fatal outcomes (Gouya et al., 2020; Souza et al., 2021). In addition, long-term complications including liver disease (cirrhosis and hepatocellular carcinoma), systemic arterial hypertension, and chronic kidney disease are part of the natural history of AHP (Gouya et al., 2020; Souza et al., 2021).

Typically, most patients with AHP are women between second and fifth decades of life with recurrent episodes of severe abdominal pain accompanied by malaise, fatigue, psychiatric disturbances (increased anxiety, inability to concentrate, and insomnia), nausea and vomiting, loss of appetite and constipation, and tachycardia (Bissell et al., 2017; Gouya et al., 2020; Souza et al., 2021). Even subtle neurological symptoms (weakness, dysesthesia, and paresthesias) lasting hours to days often require visits to emergency department and opioid prescriptions to pain relief (Bissell et al., 2017; Gouya et al., 2020; Souza et al., 2021). The dark-colored (dark brownish) urine, the clinical feature that derives the term “*porphyrus*,” may be unremarkable because ALA and PBG are colorless and is most observed after exposure of the voided urine to light, leading to the oxidative reaction of porphobilinogen to uroporphyrin and porphobilin (uroporphyrin-like pigments correlates with the typical urine color) (Bissell et al., 2017; Souza et al., 2021).

Generally, in acute attacks clinical examination is normal except for abnormal vital signs, such as tachycardia and elevated systolic blood pressure. Abdominal imaging studies exhibit non-specific findings (i.e., ileus) but are otherwise unremarkable. Laboratory evaluation is commonly normal, except for mild elevation of liver enzymes and variable hyponatremia. Most medical records of the patients disclose previous visits to the emergency department with the same symptoms and a non-diagnostic evaluation with frequent inconclusive abdominal surgical procedures and up to 15 years of interval from symptom onset until definitive diagnosis of AHP (Bissell et al., 2017; Neeleman et al., 2018; Gouya et al., 2020; Souza et al., 2021).

The acute attacks are similar for the four types of AHP but are typically more severe in AIP and ALAD deficiency. In VP and HCP, there may also be cutaneous manifestations with blisters in photoexposed skin areas, such as the face and backs of the hands and forearms (Bissell et al., 2017; Gouya et al., 2020; Souza et al., 2021). Acute neurovisceral attacks largely occur between menarche and menopause usually related to catamenial period and triggered commonly by porphyrinogenic drugs (especially oral contraceptives, barbiturates, sulfonamides, and others inducing hepatic cytochrome P450), smoking, alcohol ingestion, fasting and caloric deprivation conditions (bariatric surgery and low carbohydrate diet), emotional stress, infection, surgery, and anesthesia procedures (Bissell et al., 2017; Gouya et al., 2020; Souza et al., 2021).

A subgroup of patients presents with serious life-threatening neurological manifestations during acute attacks with peripheral motor neuropathy with quadriparesis and acute respiratory insufficiency. Seizures may be observed in up to 20% of the cases, while severe hyponatremia due to the syndrome of inappropriate antidiuretic hormone secretion (SIADH), acute encephalopathy, delirium, hallucinations, and psychosis are observed in almost 10% of the cases (Bissell et al., 2017; Souza et al., 2021). Focal neurological deficits may be associated with cerebral vasogenic edema due to complicated posterior reversible encephalopathy syndrome (PRES) (Bissell et al., 2017; Bonkovsky et al., 2019; Souza et al., 2021).

Currently, it is well established that AHP evolves with frequent and disabling chronic symptoms present in more than 65% of the patients, most of them related to neurological and psychiatric disturbances like severe chronic pain (abdominal, neuropathic, or diffuse myalgia), anxiety and mood disorders, fatigue, sleep disorders (especially insomnia), and muscle weakness with limitations for routine activities of daily living, and less than 50% of the patients reported living independently at home without special care (Bonkovsky et al., 2019; Souza et al., 2021).

## The Pathophysiology of Acute Hepatic Porphyria

**FIGURE 3:**
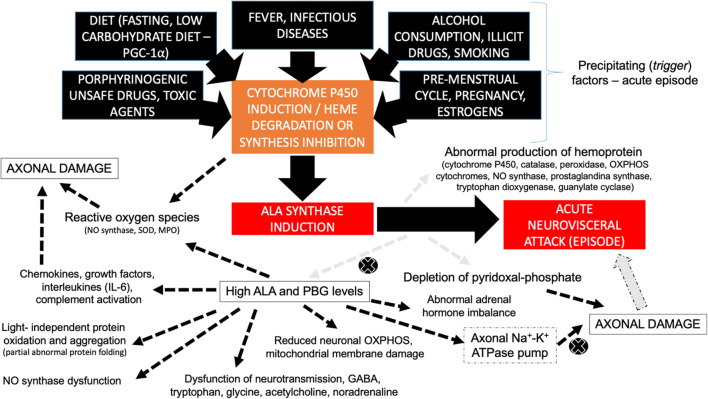
Diagram disclosing the different pathophysiological mechanisms involved with axonal damage in AHP during acute neurovisceral attacks. Different trigger (precipitating) factors lead to cytochrome P450 induction, heme group degradation, and/or the inhibition of heme synthesis, giving rise to ALA synthase induction and the secondary formation of high contents of neurotoxic ALA and PBG metabolites, leading to acute neurovisceral attack. ALA synthase induction is also associated with abnormal production of hemoprotein (related to several of the multisystemic signs and symptoms of AHP) and secondary depletion of pyridoxal-phosphate (leading to potentially reversible axonal damage). Legend, ALA, delta-aminolevulinic acid; GABA, gamma-aminobutyric acid; IL-6, interleukin-6; MPO, myeloperoxidase; NO, nitric oxide; OXPHOS, oxidative phosphorylation; PBG, porphobilinogen; PGC-1a, peroxisome proliferator-activated receptor-gamma coactivator-1 alpha; SOD, superoxide dysmutase.

A general overview discloses that several pathogenic mechanisms play an important role in AHP, especially ALA and PBG neurotoxic effects and the depletion of the heme group and hemoproteins in different tissues (Bissell et al., 2017; Bonkovsky et al., 2019; Figure 3), involving: (I) ALA-mediated dysfunction of neurotransmitter receptors particularly GABA (gamma-aminobutyric acid) that is the main inhibitory neurotransmitter in the central nervous system and GABA receptors are present in the myenteric plexus of the intestine to regulate peristalsis and muscle tone (Laiwah et al., 1983, 1985; Bonkovsky et al., 2019); (II) ALA may induce GABA-mediated inhibition of melatonin release from the pineal gland with abnormal circadian rhythm (Puy et al., 1996); (III) increased production of reactive oxygen species due to direct mitochondrial membrane compromised by persistent highly cytotoxic ALA levels, leading to chronic neurovisceral complaints and potentially to hepatic carcinogenesis (i.e., higher risk to develop hepatocellular carcinoma) (Bissell et al., 2015); (IV) modulatory effects of ALA and PBG on central and peripheral nervous system neurotransmitters and amino acid metabolism, mainly involving tryptophan, glycine, acetylcholine, and noradrenaline, especially during acute dysautonomic episodes (O’Malley et al., 2018); (V) nitric oxide synthase dysfunction with secondary vasomotor dysfunction causing cerebral and enteral vasospasm (vascular hyperreactivity) (Olivier et al., 2017; Pulgar et al., 2019); (VI) reduction of neuronal mitochondrial oxidative phosphorylation due to direct compromise of the mitochondrial membranes and the respiratory chain complexes by ALA (Ferrer et al., 2016); (VII) higher rates of mitochondrial membrane lipid oxidation due to direct damage by ALA leading to increased mitochondrial permeability (Bissell et al., 2015); (VIII) direct neurotoxic effects of ALA and PBG to the axonal membrane with dysfunction of the Na–K ATPase pump (Meyer et al., 1998; Chacko et al., 2019; Dixon et al., 2019); (IX) abnormal protein oxidation and aggregation induced by porphyrins (Maitra et al., 2015, 2019; Kupke et al., 2020); (X) depletion of pyridoxal-phosphate due to increased ALAS1 oxidative activity leading to secondary sensory axonal and small-fiber neuropathies (Hamfelt and Wetterberg, 1969); and (X) secondary effects of adrenal hormone imbalance (Pozo et al., 2014).

There is overproduction of neuroactive metabolites derived from tryptophan (especially serotonin) in AHP. Animal models showing increased plasma concentration and brain uptake of tryptophan and increased synthesis of serotonin in the central nervous system were explained due to decreased activity of tryptophan pyrrolase, which is a heme-dependent enzyme (Bonkovsky et al., 2013). This reduced activity leads to increase in serotonin plasmatic levels and can explain some of the neurovisceral features during acute attacks of porphyria (Bonkovsky et al., 2013).

Despite the suspect of possible roles for both ALA and PBG in the pathophysiology of most symptoms in all types of AHP, it is widely known that individuals harboring homozygous variants in the *HMBS* gene have severe, early, and complex presentations with marked central nervous system involvement quite distinct from classical AIP (Solis et al., 2004; Kevelam et al., 2016). So, it is a matter of concern that a distinct spectrum of disease mechanisms is involved at different stages of a disease course and in specific clinical settings, such as in early-onset cases or in cases with marked chronic intercritical symptoms (even with normal ALA and PBG levels) (Kevelam et al., 2016; Pinto, 2017).

Pathophysiological mechanisms involved with AHP have a complex association with environmental, individual, and epigenetic trigger factors (the so-called toxicogenetic disease model), such as hormonal, toxic (drugs, alcohol consumption, and plumbism), infectious, physical agents (solar exposure), dehydration, chronic inflammatory conditions of the liver, and food consumption and diet (i.e., low carbohydrate diet) (Maitra et al., 2015; Souza Júnior et al., 2017; Schmitt et al., 2018). The participation of such trigger factors has a key role in the conversion of latent AHP individuals (with oligosymptomatic or asymptomatic condition) to symptomatic-manifesting disease with variable clinical expression and potentially irreversible neuromuscular compromise (Maitra et al., 2015; Souza Júnior et al., 2017; Schmitt et al., 2018).

It is currently considered that trigger factors lead to a higher induction of P450 cytochrome with higher rates of liver metabolism activation, activation of other mitochondrial enzyme routes, and abnormal production of intermediate metabolites and modulation of hepatic ALA synthase (ALAS1), representing the main pathophysiological mechanisms of acute metabolic decompensation in AHP (Chacko et al., 2019).

## The Neuromuscular Manifestations of Acute Hepatic Porphyria and Their Pathophysiological Mechanisms

Neurological manifestations in AHP can be related to dysfunction of the central, peripheral, or autonomic nervous system. AHP should be always remembered as an important differential diagnosis for acquired and inherited neuromuscular disorders (Souza et al., 2021). In this section, we describe in detail the most common neuromuscular manifestations of AHP and the pathophysiological mechanisms involved with them.

### Acute Motor Axonal Neuropathy

Acute flaccid paralysis presenting as acute to subacute symmetric proximal quadriparesis due to motor axonal polyradiculopathy or neuronopathy represents the most common neuromuscular presentation of AHP in the Emergency Department and Intensive Care Unit (ICU), resembling clinical and neurophysiological features of Guillain–Barré syndrome and representing one of the main factors for medical and financial burden of AHP (Albers and Fink, 2004; Wu et al., 2015; Neeleman et al., 2018; Suh et al., 2019; Buendía-Martínez et al., 2021).

Classically, the peripheral neuropathy in AHP usually starts with muscle pain and weakness commonly preceded by abdominal pain and psychiatric disturbances (anxiety and insomnia) that may further progress over a 2-week period for tetraplegia or death, with some patients requiring mechanical ventilation due to paresis of respiratory and bulbar muscles. The acute motor axonal neuropathy can occur in up to 68% of patients and is usually symmetric and starts in the upper limbs with frequent association with autonomic disturbances. Cranial nerve involvement is more rarely observed, and sensory neuropathy is usually identified by mild distal lower limb paresthesias or with a painful “bathing suit” proximal distribution. Some patients may disclose permanent quadriplegia after severe acute attacks, even after proper early therapy introduction (Wikberg et al., 2000; Lin et al., 2011).

From a neurophysiological perspective, the peripheral neuropathy in AHP is characterized by reduction in compound motor action potential (CMAP) amplitude with relative preservation of conduction velocities, sensory nerve action potential (SNAP) amplitude and sensory nerve conduction velocities are usually normal or with less involvement than motor axons, and electromyography exhibit signs of acute denervation such as fibrillation potentials and fasciculations particularly in proximal muscles (Lin et al., 2011).

Neurophysiological studies are partially helpful to differentiate the Guillain–Barré syndrome (GBS) from porphyric neuropathy with the GBS presenting with delayed or absent H-reflex, delayed or absent F waves in contrast with the relative preservation of H-reflex and F wave in porphyric neuropathy, as well as prolonged distal latency and conduction block in GBS with normal latency and no conduction block in AHP (Lin et al., 2011).

Porphyrin neurotoxicity seems to occur mainly due to reduced activity of the Na^+^/K^+^-ATPase transmembrane ion pump and direct toxicity of porphyrin precursors particularly ALA and PBG; the Na^+^/K^+^-ATPase transmembrane ion pump dysfunction emerges from important mitochondrial dysfunction secondary to depletion of the heme group tissue that can lead to higher restriction of mitochondrial oxidative phosphorylation in neurons due to less efficacious hemoprotein-dependent components such as the cytochrome oxidase enzyme, and reduced ATP production by respiratory chain components may lead to abnormal axonal transport causing variable degree axonal damage and free radical overproduction (Lin et al., 2011).

There is also a major role of neurotoxic porphyrins and their intermediates in the linkage to benzodiazepine receptors of the external mitochondrial membrane coupled to voltage-gated calcium channels, leading to secondary abnormal mitochondrial oxidation (Figure 3), and it is also possible that heme biosynthesis deficiency could lead to dysfunction of proinflammatory cytokines and an increased inflammatory arm during acute decompensation (Lin et al., 2011; Wissbrock et al., 2019).

Chronic porphyric neuropathy may be observed in AHP in a different pattern of distribution with a distal sensorimotor polyneuropathy or distal motor neuropathy, even in the absence of previous neuropsychiatric disturbances or acute neurovisceral episodes, mimicking features of other peripheral neuropathies or lower motor neuron syndromes with distal and symmetric compromise, and when motor neuropathy is significant during an attack, weakness recovery slowly leaves patients with residual foot or wrist drops (Kazamel et al., 2020; Souza et al., 2021).

The motor symptoms in the context of a chronic motor axonal neuropathy in AHP are associated with irreversible damage or severe compromise to the axonal compound of the peripheral nerve after acute decompensation (Wikberg et al., 2000; Albers and Fink, 2004; Albertyn et al., 2014; Souza et al., 2018).

The pathophysiological mechanisms involved in the chronic axonal damage observed in such cases are not clear; however, in animal models with ALAS2 overexpression, it has been demonstrated that amyotrophy may result from a decrease in protein synthesis and increase in protein degradation with multiple intramyofiber dysfunction, leading to marked mitochondrial dysfunction with reduced ATP production, mitochondrial DNA content, and suppressed atrogin-1 and the skeletal E3 ubiquitin-protein ligase muscle RING-finger protein-1 (MuRF-1), which are markedly upregulated during skeletal amyotrophy denervation with sarcomere myosin heavy chain degradation (Peng et al., 2021).

The PBG deaminase-deficient animal model also discloses typical primary motor axonal degeneration with regeneration features and secondary Schwann cell reaction and mild demyelinating process (Lindberg et al., 1999).

The main differential diagnosis for acute motor axonal neuropathy seen on AHP includes acute flaccid myelitis and non-polio acute flaccid paralysis (Kaushik et al., 2014; Murphy et al., 2021).

### Acute Dysautonomia

Acute presentation of dysautonomic features in AHP is commonly observed in both pure presentation or in the typical acute neurovisceral crisis, and most patients present with peripheral autonomic involvement mainly when motor or sensory–motor axonal damage is present in acute phases or as chronic neurological manifestations (Lin et al., 2011; Kazamel et al., 2020; Souza et al., 2021).

The most common dysautonomic feature in AHP is abdominal pain, which is the most common symptom in acute attacks present in up to 80–100% of patients. Multiple pathophysiological mechanisms, such as intestinal spasm or dilatation, local vasoconstriction leading to intestinal ischemia, enteric ganglionopathy, and sensory neuropathy, may be involved (Pischik and Kauppinen, 2009; Gouya et al., 2020).

Other autonomic symptoms include tachycardia and elevated systolic blood pressure more frequent in acute attacks. Gastrointestinal complaints, including nausea, vomiting, and constipation, may be present in acute attacks as well as chronic manifestation. Furthermore, diarrhea presents during acute attacks in up to 20% of patients, while sweating disorders and fever are reported in up to 50% during episodes. Urinary bladder involvement with urinary retention or incontinence, orthostatic hypotension, and sexual dysfunction has occasionally been reported (Pischik and Kauppinen, 2009; Gouya et al., 2020; Souza et al., 2021).

Neurophysiological tests for autonomic function evaluation have been increased in neuromuscular practice, with study based on spectral analysis of heart rate variability (HRV) using low-frequency (LF) band analysis after head-up tilt table test (HUT) to evaluate cardio-sympathetic function and cardio-vagal functions analyzed by high-frequency (HF) bands during controlled breathing (12 breaths/min) and LF bands during controlled breathing (six breaths/min) in patients with AIP exhibiting decreased LF band power during deep breathing at six breaths/min compared with controls, and such difference did not occur during the HUT test suggestive of cardio-vagal impairment (Blom et al., 1996; Kazamel et al., 2020).

Some special anatomopathological features observed in autopsies of patients with AHP, such as vagus nerve demyelination, axonal loss, and neuronal cell death of sympathetic ganglion, support the direct involvement of autonomic fibers and partially explain the pandysautonomia with predominance of parasympathetic insufficiency in the natural history of AHP (Laiwah et al., 1985; Pischik and Kauppinen, 2009).

Autonomic dysfunction shares some of the same pathophysiological mechanisms involved with other central and peripheral neurological compromises observed in AHP. During acute episodes, ALA and PBG have a direct mechanism of neurotoxicity due to abnormal recapture of noradrenaline by adrenergic neurons and axonal lesion and denervation of baroreceptors (Beal et al., 1977; Laiwah et al., 1985; Lin et al., 2011). *In vitro* studies support that ALA abnormal signaling pathways through GABA receptor activation could lead to ileus symptoms observed in acute attacks (Beal et al., 1977; Laiwah et al., 1985; Lin et al., 2011).

It is also well known that serum serotonin levels and its metabolites during the changes of acute episodes may be abnormal, providing similar mechanisms to that observed in other contexts of serotoninergic syndromes and the imbalance of the kynurenine metabolic pathway (a key tryptophan related metabolite) with increase in the urinary kynurenine/tryptophan ratio suggests that a secondary induction of liver indoleamine 2,3-deoxygenase may play a role in tryptophan and serotonin abnormalities in AHP (Bonkovsky et al., 2013; Gomez-Gomez et al., 2017).

The reduction of tryptophan pyrrolase activity in other stages of acute episode presentation leads to low serotonin and tryptophan contents and is also an important contributing mechanism previously demonstrated in rat models (Badawy, 1978). It is also possible that similar mechanisms to that linked to vascular hyperreactivity may also play an important pathophysiological role in dysautonomia (Pulgar et al., 2019).

### Rhabdomyolysis

Acute rhabdomyolysis has been described several times associated with acute neurovisceral crisis in AHP, most commonly in the context of severe presentations in the ICU with hydroelectrolytic disturbances and preceded by autonomic neuropathy and acute encephalopathy (Devars du Mayne et al., 1987; Marsden and Peters, 2004; García-Martul et al., 2008; Yrjönen et al., 2008; Jaramillo-Calle and Acevedo, 2019). Isolated or recurrent episodes of rhabdomyolysis are not typically observed in AHP (Adams and Amaya, 2014), and it is not usual to represent the presenting clinical event, and myoglobinuria is a variable finding (Yrjönen et al., 2008; Adams and Amaya, 2014; Karakulova et al., 2019). The pathophysiological basis is not completely understood, despite some possible hypotheses suggesting that increased ALA levels in skeletal muscle tissue represent a potential myotoxic damaging metabolite and the possibility of ischemic muscle damage in a similar pattern to that observed with sustained cerebrovascular vasospasm due to arteriolar dysfunction linked to vasoconstriction properties of ALA (Marsden and Peters, 2004; García-Martul et al., 2008; Olivier et al., 2017). Deficiency of the heme-dependent nitric oxide synthase leads to reduced nitric oxide production, resulting in abnormal vasoregulation of muscle arterioles and increased platelet activation due to reduced guanylate cyclase activity and, thus, leading to ischemic muscle injury (Marsden and Peters, 2004; García-Martul et al., 2008; Olivier et al., 2017).

Hyponatremia-induced muscle necrosis during acute neurovisceral attacks has been observed in contexts with SIADH or inappropriate hyponatremia correction (García-Martul et al., 2008; Chen et al., 2015). The intracellular potassium efflux and marked osmolarity changes, and the reduction of muscle transmembrane potentials and decreased skeletal muscle metabolism contribute to rhabdomyolysis in this context (García-Martul et al., 2008; Chen et al., 2015). Furthermore, patients with acute neuropsychiatric presentation with psychosis or convulsive status epilepticus can disclose periods of intense isometric and sustained muscle contraction, contributing to the development of acute rhabdomyolysis (Marsden and Peters, 2004).

### Painful Small-Fiber Neuropathy

Chronic pain represents one of the most common clinical complaints of patients with AHP during the disease course, regardless of the severity or recurrence frequency of previous acute neurovisceral attacks (Gouya et al., 2020; Souza et al., 2021).

Tickling, paresthesias, allodynia, and numbness are commonly observed as chronic neuromuscular compromise as well as chronic axonal sensory neuropathy signs (Cardenas and Guerrero, 2018; Vita et al., 2019; Sachau et al., 2021). The acute and chronic neurotoxic effect of abnormally raised levels of heme precursors ALA and PBG seems to represent the main pathophysiological mechanism associated with small-fiber neuropathy (Sachau et al., 2021). Abnormal central and peripheral sensitization phenomena occur probably due to the increased brain tryptophan levels resulting from raised 5-hydroxy-tryptamine catabolism and decreased activity of liver tryptophan pyrrolase trough nerve pathways that are linked to the thalamus and cortex regions responsible for nociceptive information and pain control (Cardenas and Guerrero, 2018; Sachau et al., 2021). Most patients do not have marked sensory loss on examination, even in the presence of focal peripheral demyelination, and alterations in thermal sensitivity and muscle pain are suggestive of damage to the small sensory nerve fibers, which have been confirmed by a marked decrease in the density of intra-epidermal nerve fibers in skin biopsies (Cardenas and Guerrero, 2018; Sachau et al., 2021).

Chronic and acute neuropathic pain and axonal damage may be at least, in part, due to the higher activity of ALAS1 during acute decompensation and in recurrent contact with trigger factors leading to abnormal glycine consumption and reduction of acetylcholine biosynthesis in presynaptic terminals, and there is also an undefined impact role of reduced acetylcholine release in the neuromuscular junction mediated by direct effect of ALA and PBG in the pathophysiology of neuropathic symptoms and signs, similar to previous evidence linked to the inhibition of cholinesterases in several brain regions (Rodriguez et al., 2002).

### Chronic Fatigue

Chronic fatigue represents one of the most important chronic symptoms in AHP leading to decreased quality of life, mainly in patients with recurrent life-threatening acute neurovisceral attacks (Naik et al., 2016; Gouya et al., 2020). However, oligosymptomatic and non-recurrent attack patients may present with chronic fatigue, possibly not associated with previous muscle or nerve toxic damage mechanisms (Buendía-Martínez et al., 2021). Porphyrins are considered to have a possible key role involved in several fatigue syndromes; however a well-defined mechanism related to fatigue pathogenesis in AHP is still unknown (Downey, 1994). It is unknown if the previously discussed mechanisms involving mitochondrial dysfunction, abnormal hemoprotein compound formation, and direct muscle injury may play any role in its pathogenesis (Lin et al., 2011; Wissbrock et al., 2019; Buendía-Martínez et al., 2021). There is also a large discussion regarding the possible role of chronic neuropathic painful symptoms and chronic or recurrent sleep disturbances in the emergence of chronic fatigue, so that both affects up to 15% of all AHP patients at the late stages of a disease course (Gouya et al., 2020; Souza et al., 2021).

## Genetic Basis of Acute Hepatic Porphyria

**FIGURE 4:**
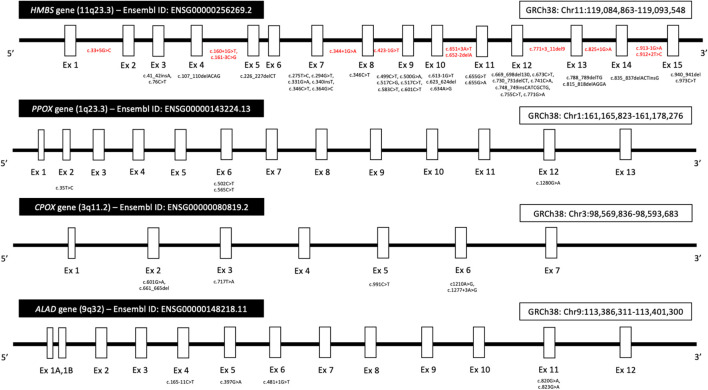
Diagram showing the location of pathogenic and likely pathogenic variants in the four genes (*HMBS*, *PPOX*, *CPOX*, and *ALAD*) associated with AHP. Variants were included according to data from the Genome Aggregation Database (gnomAD, browser v2.1.1), the Ensembl Project browser, and the ClinVar database public archive (all last accessed on August 10, 2021). Most variants in exon regions (black color) are missense. Intronic variants are represented in red color. Legend: Ex, exon; GRCh, human Genome Reference Consortium.

Acute hepatic porphyria represents a classical group of inherited metabolic disease with incomplete penetrance and large intrafamilial expressivity (Bissell et al., 2017; Souza et al., 2021). Phenotypic expression results in AHP from a complex interaction between an individual monogenic basis (the involved pathogenic variant in AHP-related genes and other possible genetic modulation mechanisms) and epigenetic and environmental factors (O’Malley et al., 2018; Kazamel et al., 2020; Souza et al., 2021), as previously discussed. A summary of the main pathogenic and likely pathogenic variants reported to date in the four genes associated with AHP is provided (Figure 4). Most pathogenic variants related to AHP are classified as missense, small deletions, and small insertions or duplications (Chen et al., 2016; O’Malley et al., 2018; Jaramillo-Calle and Acevedo, 2019). AIP, VP, and most cases of HCP are associated with autosomal dominant pattern of inheritance and occurs due to heterozygous pathogenic variants, respectively, in *HMBS*, *PPOX*, and *CPOX* genes. Biallelic variants have been also described rarely in HCP and its extremely rare variant presentation called harderoporphyria. Homozygous and compound heterozygous variants in *ALAD* gene are also identified in the rare context of autosomal recessive Doss porphyria (ALAD deficiency). Compound heterozygous variants have also been identified in rare presentations of childhood-onset autosomal recessive AIP (O’Malley et al., 2018; Jaramillo-Calle and Acevedo, 2019; Souza et al., 2021).

Molecular genetic studies are currently considered the gold standard method to confirm a highly suspected clinical case of AHP (especially during intercritical periods and in individuals with unremarkable biochemical evaluation) or in patients with suggestive metabolic profiles from qualitative, semiquantitative, or quantitative measurement assay for urine PBG. Gene sequence analysis (single-gene-based testing) and next-generation sequencing (NGS) with gene panel testing for AHP represent the most used approaches in clinical practice (O’Malley et al., 2018; Wang et al., 2018; Kazamel et al., 2020; Souza et al., 2021).

## Clinical Management and Therapeutic Approaches

**FIGURE 5:**
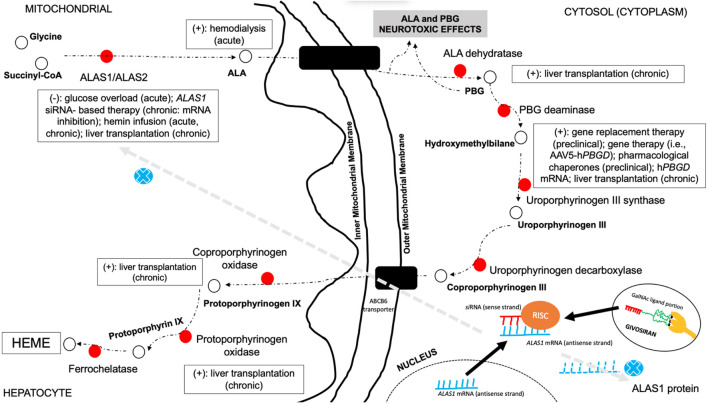
Possible therapeutic approaches in the management of AHP and their associated mechanism of action in the multistep pathway of heme biosynthesis. This figure highlights the different steps of heme biosynthesis pathway in which different therapy options may be used to treat AHP. Glucose overload and hemin-based therapies are directed to downregulate hepatic ALAS1 production and reducing, thus, heme biosynthesis pathway activation, limiting neurotoxic metabolite production. Hemodialysis represents an option to withdraw neurotoxic metabolites from circulation in acute severe presentation without available hemin-based therapy. Pharmacological chaperone aids in the proper function of misfolded enzymes and directing properly to the targeted route. Gene therapy approaches are directed to provide liver production of the specific deficient enzyme of the pathway. Small interfering RNA-based therapies are targeted to hepatocyte delivery, promoting association with the antisense strand of *ALAS1* mRNA and the formation of RISC in the cytosol and then inhibiting ALAS1 production. Liver or hepatocyte transplantation enables the restoration of normal functioning enzyme in different types of AHP. Legends: AAV5, adeno-associated virus type 5; ALA, delta-aminolevulinic acid; ALAS, delta-aminolevulinic acid synthase; CoA, coenzyme A; GalNAc, *N*-acetylgalactosamine; PBG, porphobilinogen; PBGD, porphobilinogen deaminase; RISC, RNA-induced silencing complex; siRNA, small interfering RNA; (+), treatment option for the step—normal enzyme activity; (–), treatment option associated with inhibition of enzyme production.

Clinical management of AHP is complex and is based on an individual-based approach and has been widely discussed in previous literature (Balwani et al., 2017; Anderson, 2019; Fontanellas et al., 2019; Zhao et al., 2020; Bustad et al., 2021; Souza et al., 2021; Figure 5). Most therapeutic measures currently used in the treatment of patients with AHP have been developed based on the expanding knowledge of heme biosynthesis group inhibitor or activator triggers, as well as in new regulators of metabolites or transcription factors involved with different enzyme steps (Balwani et al., 2017; Anderson, 2019; Fontanellas et al., 2019; Zhao et al., 2020; Bustad et al., 2021). There are three different periods and approaches during the treatment of AHP for the proper management of acute and chronic complications of the disease (Anderson and Collins, 2006; Anderson, 2019). First, during acute neurovisceral attacks, it is recommended to screen the patient for potentially identifiable and treatable trigger factors (i.e., withdrawal of porphyrinogenic drugs and infectious diseases), classify the severity and possible outcomes of the presentation (Souza et al., 2021), and provide specific therapies for the treatment, such as glucose overload therapy (high doses of glucose infusion) in mild episodes (or as a transient therapy in cases without ready availability of other specific therapies) and hemin-based therapies for moderate and severe presentation (i.e., hematin and heme arginate) (Anderson and Collins, 2006; Anderson, 2019; Souza et al., 2021). In cases with severe presentation and no specific therapies available, hemodialysis may also be used as an exceptional life-saving measure, and liver transplantation has been previously reported in small case series as a possible successful treatment for patients with recurrent attacks (Anderson and Collins, 2006; Anderson, 2019; Lissing et al., 2021; Souza et al., 2021).

Hemin-based therapies represent the most classical therapeutic approach for acute neurovisceral attacks in AHP and are mainly indicated for moderate-to-severe presentation or in mild cases treated with high glucose overload without clinical improvement (refractory mild attacks). Scheduled infusions of hemin-based therapies may be also used in the treatment of selected patients with recurrent non-cyclical acute attacks and are most used biweekly or weekly (Anderson and Collins, 2006; Souza et al., 2021). Hemin groups taken by hepatocytes after infusion are oxidized iron protoporphyrin IX molecules, which promote downregulation of ALAS1 biosynthesis and, thus, inhibition of neurotoxic porphyrin production, leading to progressive clinical improvement of neurological compromise. Both presentations as heme arginate and hematin are currently available in several countries and represent the most widely used specific therapy for AHP attacks. Hematin (Panhematin^®^) can be used during attacks with a dose of 3–4 mg/kg per day, for 4–5 days each cycle. Heme arginate (Normosang^®^) can be used during attacks with a dose of 3 mg/kg per day (up to 250 mg per day) for 4 days. Both presentations need careful clinical and laboratorial monitoring during infusion periods, and different chronic and acute adverse events have been correlated with them (Anderson and Collins, 2006; Balwani et al., 2017; Anderson, 2019; Fontanellas et al., 2019; Zhao et al., 2020; Bustad et al., 2021; Souza et al., 2021).

Long-acting gonadotropin-releasing hormone receptor or luteinizing hormone-releasing hormone (GnRH/LH-RH) agonists have an important contribution in the management of patients with severe recurrence during premenstrual periods or cyclical attacks (Schulenburg-Brand et al., 2017). In cases without good clinical stabilization of attack recurrence, even after proper treatment, liver transplantation has been performed by some centers, mainly for patients with AIP. There is no recognizable difference in the general rates of complications, retransplantation, or survival after transplantation in patients with AIP compared with other inherited metabolic disorders. The worst outcomes and the highest rates of complications are observed in patients with previously established severe neurological compromise and in some individuals with chronic kidney disease (Lissing et al., 2021). Combined kidney and liver transplantation has been rarely performed in AHP, mainly in patients with severe metabolic decompensation and chronic kidney disease as a serious complication in late-stage disease course (Lissing et al., 2021). There is still low evidence to propose allogeneic hepatocyte transplantation rather than traditional liver transplantation for AIP, despite some studies with the *Hmbs*-deficient mouse model showing important reduction in ALA and PBG plasma levels (Lissing et al., 2021).

Currently, there are few options for the treatment of patients with recurrent attacks of AHP with hemin-based therapies approved just for the management of acute attacks and without scientific evidence to be used as a preventive therapeutic option as well as is associated with worrisome life-threatening events associated with repeated administrations including increased risk of chronic liver inflammation, secondary hemochromatosis, acute tubular necrosis, tachyphylaxis, and thrombophlebitis (Yarra et al., 2019).

More recently, small interfering RNA (siRNA)-based therapies for AHP (givosiran, an *N*-acetyl-D-galactosamine-conjugated siRNA) (Springer and Dowdy, 2018) have been studied and provided major results in the control of patient recurrence rates and without established severe adverse events during infusion or after chronic use (Figure 5; Balwani et al., 2017, 2020; Sardh et al., 2019; Vita et al., 2019; De Paula Brandão et al., 2020).

Givosiran (Givlaari^TM^) is a synthetic double-stranded siRNA covalently linked to a ligand containing three *N*-acetylgalactosamine (GalNAc) residues that bind with high affinity to asialoglycoprotein receptors (ASGPRs), exclusively expressed on hepatocytes and specifically targets ALA synthase 1 (*ALAS1*) messenger RNA in the liver with downregulation on its elevated levels and reducing circulating levels of neurotoxic metabolites ALA and PBG (Blouin et al., 2013). The interaction between ASPGR and givosiran leads to a membrane pore formation with cellular uptake of siRNA into endosomes that will suffer pH changes with subsequent release of siRNA to the cytoplasm where double strand is recognized by a protein complex, including DICER protein (a ribonuclease protein), TRBP (TAR RNA-binding protein) and PACT (protein activator of interferon-induced protein kinase, PKR protein activator), which eliminates the sense strand for degradation, while the guided strand remains linked giving rise to the RISC complex (RNA-induced silencing complex) that contains the argonaute protein (AGO), and this guide strand will conduct the RISC complex to mRNA that holds the complementary sequence, and the AGO enzyme executes *ALAS1*–mRNA cleavage interrupting heme biosynthesis (Springer and Dowdy, 2018; De Paula Brandão et al., 2020).

The safety and efficacy of givosiran was evaluated in a randomized double-blind, placebo-controlled, phase 3 trial (ENVISION, NCT03338816) with enrolled 94 patients that had definitive diagnosis of AHP and met the inclusion criteria (age ≥ 12 years old and having a minimum of two attacks requiring hospitalization, urgent healthcare visits, or intravenous hemin use in the 6 months prior to study entry 77). The primary endpoint was the annualized rate of composite porphyria attacks (number of attacks requiring hospitalization, urgent care visit, or home intravenous heme infusion at 6 months), and key secondary endpoints were ALA and PBG levels, number of hemin doses, specific symptoms (pain, fatigue, and nausea), and quality of life measure by PCS of SF-12 questionnaire (Balwani et al., 2020). Of the 94 randomized patients, 48 were assigned to receive once-monthly subcutaneous givosiran at 2.5 mg/kg dose, and 46 went to the placebo arm with final results showing that the mean annualized attack rate (AAR) was 3.2 in the givosiran group and 12.5 in the placebo group, representing a 74% lower rate in the givosiran group (*p* < 0.001); at 6 months, the median composite AAR was reduced by 90% in givosiran patients compared with placebo (median composite AAR 10.7 vs. 1.0), and there was a threefold increase in the percentage of patients who were attack free at the givosiran group (50 vs. 16.3% at placebo group). The analysis of secondary endpoints showed that mean ALA and PBG levels were reduced from baseline by 77 and 76% with *p* < 0.001, respectively, in the givosiran group, and the mean number of days of hemin use was also significantly reduced in patients treated with givosiran compared with placebo (4.7 vs. 12.8 days, *p* = 0.0002). Regarding safety data, the most common adverse reactions occurring at least 5% more frequently in patients receiving givosiran than in placebo were nausea (27 vs. 11%), injection site reactions (25 vs. 0%), rash (17 vs. 4%), serum creatinine increase (15 vs. 4%), transaminase elevations (13 vs. 2%), and fatigue (10 vs. 4%) with just one patient discontinuing givosiran treatment because of transaminase elevations (Balwani et al., 2020). Nowadays, givosiran (Givlaari^TM^) is the only pharmacological treatment approved by the FDA, EMA, and other worldwide health agencies for the treatment of adult patients with AHP.

New therapeutic approaches with special focus on enzyme stabilization and the modulation of proteostasis regulators using pharmacological chaperone therapy, and ubiquitin–proteasome inhibitors are underway and may represent new prophylactic therapies (Bustad et al., 2021). Regarding the use of chaperone and proteasome inhibitor agents, there is currently no evidence of clinical benefit of therapeutic use of proteasome inhibitors (i.e., bortezomib) in AHP. Bortezomib has been evaluated in experimental studies involving congenital erythropoietic porphyria mouse mutants (UROS^C73R^ and UROS^P248Q^) and provided marked reduction in photosensitivity skin lesions and in porphyrin excretion in urine or accumulation in erythrocytes (Blouin et al., 2013). Pharmacological chaperones are under investigation for AIP and have been demonstrated in *Hmbs*-deficient mouse model potential benefit in the reduction of several neurotoxic porphyrin precursors due to the targeted stabilization of unstable and improperly folded HMBS enzyme (Bustad et al., 2021).

Regarding other therapy approaches, gene replacement therapy and, more recently, recombinant adeno-associated viral vector (rAAV2/8) gene therapies are currently under investigation in experimental models and early stages of clinical trials. After more than a decade after the first experimental studies started, there is special concern regarding increasing vector doses in different genetic conditions and potential harmful and life-threatening adverse events. However, a successful attempt to provide a hyperfunctional porphobilinogen deaminase enzyme in a mouse model of AIP and the lower AAV vector dose disclosed a more safety approach at that time (Serrano-Mendioroz et al., 2018). The first phase I open-label multicenter clinical trial was performed using a rAAV2/5 vector for the overexpression of porphobilinogen deaminase in patients with AIP. Despite the safe and initial positive effect on clinical outcomes at this stage (i.e., reduced number of hospital admissions and hemin therapy use), there was no significant changes in ALA and PBG levels during follow-up. The development of anti-AAV5 neutralizing antibodies after treatment and the absence of marked reduction in neurotoxic metabolites still represent a challenge for future phases of the trial and for other potential molecule candidates (D’Avola et al., 2016). Traditional intravenous enzyme replacement therapies with the aim to provide higher levels of functional enzyme to degrade the neurotoxic amounts of ALA and PBG were evaluated previously without significant changes in clinical outcomes, despite the demonstration of reduced concentrations of plasma ALA and PBG (Bustad et al., 2021).

## Conclusion

The current knowledge about the pathophysiological mechanisms involved with neuromuscular manifestations of AHP is expanding and discloses the complex interface of multiple intracellular pathways and the potential role of abnormal intermediate products and metabolites. More specific and targeted therapies based on new gene therapies and siRNA-based platforms will probably provide better quality of life, reduction in the relapsing rates of acute neurovisceral episodes, and improve chronic neuromuscular complaints associated with AHP, such as chronic fatigue and painful small-fiber neuropathy.

## Author Contributions

All authors listed have made a substantial, direct and intellectual contribution to the work, and approved it for publication.

## Conflict of Interest

PS had received honorarium from Alnylam Pharmaceuticals as speaker and scientific advisory boarding member. The remaining authors declare that the research was conducted in the absence of any commercial or financial relationships that could be construed as a potential conflict of interest.

## Publisher’s Note

All claims expressed in this article are solely those of the authors and do not necessarily represent those of their affiliated organizations, or those of the publisher, the editors and the reviewers. Any product that may be evaluated in this article, or claim that may be made by its manufacturer, is not guaranteed or endorsed by the publisher.
